# Dose‐Related Gastric Toxicity of Diethyl Phthalate in Female Wistar Rats

**DOI:** 10.1002/jat.70445

**Published:** 2026-09-17

**Authors:** Gul Sahika Gokdemir, Mehmet Cihan Yavaş, Selim Demirtaş, Fazile Cantürk Tan, Salih Varol, Mehmet Tahir Gokdemir

**Affiliations:** ^1^ Faculty of Medicine, Department of Physiology Mardin Artuklu University Mardin Turkey; ^2^ Faculty of Medicine, Department of Biophysics Mardin Artuklu University Mardin Turkey; ^3^ Faculty of Medicine, Department of Histology and Embryology Mardin Artuklu University Mardin Turkey; ^4^ Faculty of Medicine, Department of Biophysics Erciyes University Kayseri Turkey; ^5^ Faculty of Medicine, Department of Emergency Medicine Mardin Artuklu University Mardin Turkey

**Keywords:** apoptosis, diethyl phthalate, DNA damage, gastric mucosa, gastric mucosal defense, oxidative stress

## Abstract

Diethyl phthalate (DEP) is commonly used in cosmetics, personal care products, and pharmaceutical formulations as a low‐molecular‐weight phthalate. Despite its widespread use, information on its effects on the gastric mucosa remains limited. This study examined the dose‐related effects of DEP on inflammation, oxidative stress, gastric mucosal defense, DNA damage, apoptosis, and histopathological changes in rat gastric tissue. Twenty‐eight female Wistar rats were randomly assigned to four groups (*n* = 7 per group): control and DEP (100, 300, or 600 mg/kg/day). These experimental toxicological doses were administered by oral gavage for 21 days. Gastric tissue levels of interleukin‐6 (IL‐6), tumor necrosis factor‐α (TNF‐α), cyclooxygenase‐2 (COX‐2), superoxide dismutase (SOD), malondialdehyde (MDA), trefoil factor family 1 (TFF1), and mucin‐5AC (MUC5AC) were determined by ELISA. Histopathological examination, Bax/Bcl‐2 immunohistochemistry, and comet assay were performed. Dose–response relationships were assessed using polynomial trend analysis. DEP exposure resulted in significant dose‐dependent increases in IL‐6, TNF‐α, COX‐2, MDA, Bax expression, Bax/Bcl‐2 ratio, histopathological damage, and DNA damage (all *p* < 0.001), whereas SOD levels and Bcl‐2 expression significantly decreased (all *p* < 0.001). TFF1 and MUC5AC levels were significantly increased at 100 mg/kg but significantly decreased at 600 mg/kg compared with controls. Histopathological examination showed progressive gastric mucosal injury with increasing DEP doses. DEP exposure induced dose‐related changes in gastric inflammation, oxidative stress, mucosal protective factors, histopathology, DNA damage, and apoptosis‐related protein expression. The findings suggest that oxidative stress and inflammation are associated with altered mucosal defense, DNA damage, and apoptosis following DEP exposure.

## Abstract 2

Diethyl phthalate caused dose‐related gastric injury in female Wistar rats. DEP exposure increased inflammatory and oxidative stress markers, altered TFF1 and MUC5AC levels, and was associated with DNA damage, apoptosis‐related protein changes, and progressive histopathological injury.

## 1 Introduction

Phthalates are used in plastics and a variety of consumer products, including cosmetics, pharmaceuticals, food packaging, and personal care products. Because they are not permanently bound to polymers, they can be released during production and use. Human exposure occurs through food, drinking water, indoor air, and household dust (Giuliani et al. 2020; Serrano et al. 2014; Wang et al. 2019). Phthalates and their metabolites have been detected in human urine, serum, and breast milk, indicating widespread exposure to this chemical group (Wang et al. 2019).

Diethyl phthalate (DEP) is a low‐molecular‐weight phthalate commonly used as a solvent and fragrance stabilizer. It is found in cosmetics, perfumes, personal care products, and several pharmaceutical formulations (Wang et al. 2019). Although DEP has generally demonstrated lower toxicity than some higher‐molecular‐weight phthalates in several experimental models, evidence indicates that repeated exposure can affect multiple biological pathways (Weaver et al. 2020). Experimental studies have associated DEP exposure with oxidative stress and inflammatory responses, while apoptotic changes have also been reported in different tissues and experimental models (Kim et al. 2025; Mondal et al. 2019; Weaver et al. 2020).

Most experimental studies have focused on the effects of phthalates on the liver, kidneys, endocrine organs, and reproductive tissues (Giuliani et al. 2020; Weaver et al. 2020). Following oral intake, DEP enters the digestive tract, where it is hydrolyzed to monoethyl phthalate (MEP), its major metabolite. MEP is commonly measured in urine as a biomarker of DEP exposure, although the extent of DEP hydrolysis within the human digestive tract has not been fully established (Sekizawa et al. 2003; Wang et al. 2019). Despite this gastrointestinal exposure, information on the gastric effects of DEP remains limited. An early dietary study reported increased absolute and relative weights of the stomach and small intestine in DEP‐exposed rats, with significant increases observed in females at all dietary levels after 16 weeks; however, no histological changes were detected (Brown et al. 1978). To our knowledge, gastric oxidative stress, inflammatory markers, and DNA damage have not previously been examined following DEP exposure. The gastric mucosa is continuously exposed to food components and exogenous contaminants, including phthalates present in food and drinking water, and its defense relies on the coordinated function of epithelial cells, the mucus layer, and protective mucosal proteins (Giuliani et al. 2020; Serrano et al. 2014). Inflammation and oxidative stress can weaken mucosal defense and cause epithelial injury. Damage to the mucosa may, in turn, further increase local inflammatory and oxidative responses (Heuer et al. 2020; Hoffmann 2020).

Gastric mucosal injury develops when damaging factors exceed the capacity of local protective mechanisms. Increased reactive oxygen species production and lipid peroxidation, together with reduced antioxidant activity, can damage epithelial cells and intensify local inflammatory responses. Increased production of inflammatory cytokines, including TNF‐α and IL‐6, may further impair mucus‐dependent defense and epithelial integrity (Ahmed, Alqaisi, Ibrahim, et al. 2025; Al‐Qaisi et al. 2025). Changes in apoptosis‐related proteins, including Bax and Bcl‐2, may also contribute to the loss of gastric epithelial cells (Ahmed, Alqaisi, Jabbar, et al. 2025; Ahmed, Alqaisi, et al. 2026; Ahmed, Tawfeeq, et al. 2026). These processes have been reported together in experimental models of gastric mucosal injury and ulceration, although the initiating agents and experimental conditions differ among models.

Trefoil factor family 1 (TFF1) and mucin 5AC (MUC5AC) are important components of gastric mucosal protection. TFF1 supports epithelial repair after injury and contributes to mucosal healing. MUC5AC is the major gel‐forming mucin in the stomach and forms a physical barrier against luminal irritants. Reduced expression of either molecule weakens mucosal defense and increases tissue susceptibility to injury. Oxidative stress and inflammatory injury can affect gastric surface mucous cells and mucus production. Because TFF1 is co‐secreted with MUC5AC by these cells, DEP‐associated oxidative stress and inflammation may affect both mucosal protective factors (Breugelmans et al. 2022; Heuer et al. 2020; Hoffmann 2020). To our knowledge, the effects of DEP or other phthalates on gastric TFF1 and MUC5AC have not been reported.

Gastric inflammation, oxidative stress, TFF1 and MUC5AC levels, histopathological injury, DNA damage, and apoptosis‐related protein changes have not previously been evaluated together following DEP exposure. Examining these outcomes in the same experimental model may provide a more complete understanding of DEP‐induced gastric injury.

Therefore, this study examined whether increasing DEP doses cause gastric injury in female Wistar rats and whether the observed responses follow linear or nonlinear dose‐related patterns. Because mucosal protective responses might initially increase but decline as tissue injury becomes more severe, polynomial trend analysis was used to evaluate both patterns. We hypothesized that DEP exposure would induce gastric injury associated with inflammation, oxidative stress, altered mucosal defense, DNA damage, apoptosis‐related protein changes, and histopathological alterations.

## 2 Materials and Methods

### 2.1 Animals and Experimental Design

Twenty‐eight female Wistar albino rats (8–10 weeks old, 180–200 g) were included in this study. Female rats were selected because perfumes and fragranced personal care products are important sources of DEP exposure among women (Parlett et al. 2013; Wang et al. 2019). The animals were randomly assigned to four experimental groups (*n* = 7 per group).

DEP (≥ 99% purity, Sigma‐Aldrich, St. Louis, MO, USA) was dissolved in corn oil immediately before administration and given once daily by oral gavage for 21 consecutive days. All groups received a fixed gavage volume of 0.5 mL/day. Body weight was checked daily, and the amount of DEP administered to each rat was adjusted accordingly to provide doses of 100, 300, or 600 mg/kg/day within the fixed 0.5‐mL volume. Administration was performed at approximately the same time each morning. Animals were not fasted before administration. Rats in the control group received corn oil alone at the same volume and by the same route.

The doses were selected based on previous repeated‐dose oral DEP toxicity studies in rodents, which used experimental doses extending up to 1000 mg/kg/day (Sekizawa et al. 2003; Shiraishi et al. 2006; Weaver et al. 2020). The three doses were chosen to examine gastric responses across a range of non‐lethal experimental exposures. The doses were not derived from human exposure estimates or NOAEL/LOAEL values, and no pilot dose‐ranging study was performed.

The experimental groups were defined as follows:

 
Control: corn oil (0.5 mL/day)
 
DEP‐100: DEP 100 mg/kg/day
 
DEP‐300: DEP 300 mg/kg/day
 
DEP‐600: DEP 600 mg/kg/day

The present study was a secondary analysis of previously unused gastric tissues collected at sacrifice from the same cohort of animals used in previous studies (Kömürcü and Yavaş 2026; Yavaş et al. 2026). The previous studies evaluated thyroid, liver, and serum outcomes. The collection of gastric tissues at sacrifice was included in the original experimental protocol, whereas their use for the present analyses was subsequently approved by the ethics committee. No gastric biochemical, histopathological, immunohistochemical, or comet assay data from the present study have been published previously, and no additional animals were used. The group size was predetermined by the original animal experiment. Because the present study used previously unused gastric tissues from that cohort, no separate a priori sample‐size calculation was performed for the gastric outcomes. A sensitivity analysis based on four groups and 28 animals indicated that the study had 80% power to detect an overall effect size of Cohen's *f* ≥ 0.68 at *α* = 0.05.

All animals were maintained under standard laboratory conditions with free access to standard chow and tap water. The animal room was maintained at 23°C ± 2°C and 45%–55% relative humidity, with a 12‐h light/12‐h dark cycle.

### 2.2 Ethics Approval

The original animal experiment was approved by the Local Ethics Committee for Animal Experiments of Dicle University Health Sciences Research and Application Center (Protocol No. 2024/34). Ethical approval for the use of previously unused gastric tissues from these animals in the present study was obtained from the same committee (Meeting No. 02, Decision No. 18; January 29, 2026). All procedures complied with national regulations for laboratory animal care and followed the recommendations of the National Research Council Guide for the Care and Use of Laboratory Animals. Reporting of the study followed the ARRIVE 2.0 guidelines (Percie du Sert et al. 2020).

### 2.3 Sample Collection

At the end of the 21‐day exposure period, anesthesia was induced with ketamine hydrochloride (90 mg/kg) and xylazine (9 mg/kg) administered intraperitoneally. After adequate anesthesia had been confirmed, the animals were euthanized by exsanguination. The stomach was removed immediately and washed gently with cold phosphate‐buffered saline (PBS). Samples intended for biochemical analyses were frozen at −80°C until use. Tissue specimens for histopathological and immunohistochemical analyses were fixed in 10% neutral‐buffered formalin.

### 2.4 Biochemical Analyses

Frozen gastric tissues were thawed on the day of analysis and weighed individually. Each sample was homogenized in ice‐cold PBS (pH 7.2) at a tissue‐to‐buffer ratio of 1:10 (w/v) using a tissue homogenizer (ISOLAB, Germany) (Guzmán‐Gómez et al. 2023). The homogenates were centrifuged at 12,000 × g for 12 min at 4°C, and the supernatants were collected for biochemical analyses.

Gastric tissue levels of interleukin‐6 (IL‐6; SunRed Biotechnology, Shanghai, China; Cat. No. 201‐11‐0136), tumor necrosis factor‐α (TNF‐α; SunRed Biotechnology; Cat. No. 201‐11‐0765), cyclooxygenase‐2 (COX‐2; SunRed Biotechnology; Cat. No. 201‐11‐0297), superoxide dismutase (SOD; YL Biont, Shanghai, China; Cat. No. YLA0115RA), malondialdehyde (MDA; YL Biont; Cat. No. YLA0029RA), trefoil factor 1 (TFF1; SunRed Biotechnology; Cat. No. 201‐11‐2320), and mucin 5AC (MUC5AC; SunRed Biotechnology; Cat. No. 201‐11‐0749) were measured using commercially available rat‐specific ELISA kits. According to the manufacturers, the kits were suitable for rat tissue homogenates; however, no separate validation specifically using rat gastric tissue was performed. All assays were performed according to the manufacturers' instructions, and all samples were analyzed in duplicate.

A pilot analysis was performed to determine whether additional sample dilution was required. Based on the pilot measurements, all samples used for the MDA, IL‐6, and TNF‐α assays were diluted 1:10 before analysis. Concentrations calculated from the standard curves were multiplied by the dilution factor, and the dilution‐corrected concentrations were used in the statistical analyses and are reported in the tables. All other analytes were measured without further dilution.

The analytical sensitivities and measurement ranges specified by the manufacturers were 1.822 pg./mL and 2–600 pg./mL for IL‐6, 5.127 ng/L and 8–1000 ng/L for TNF‐α, 0.446 ng/mL and 0.5–150 ng/mL for COX‐2, 0.016 ng/mL and 0.05–20 ng/mL for SOD, 0.024 nmol/mL and 0.05–10 nmol/mL for MDA, 0.112 ng/mL and 0.15–40 ng/mL for TFF1, and 0.072 ng/mL and 0.1–20 ng/mL for MUC5AC, respectively. The intra‐ and inter‐assay coefficients of variation were below 9% and 11%, respectively, for the SunRed assays and below 8% and 10%, respectively, for the YL Biont assays.

Concentrations were calculated from the corresponding standard curves and expressed per milliliter of tissue homogenate supernatant prepared using a standardized tissue‐to‐buffer ratio of 1:10 (w/v). No normalization to total protein content was performed. Absorbance was measured at 450 nm using a Biochrom Anthos Zenyth 200 microplate reader (Biochrom Ltd., Cambridge, UK).

### 2.5 Histopathological Examination

Paraffin‐embedded gastric tissue samples were cut into 5‐μm sections and stained with hematoxylin and eosin (H&E) according to routine histological procedures. The stained sections were examined using a light microscope (Carl Zeiss Axiolab 5, Carl Zeiss, Oberkochen, Germany). Representative images were captured and analyzed with ZEN 3.7 software (Carl Zeiss, Oberkochen, Germany).

Histopathological examination was performed to evaluate gastric injury following DEP exposure. Tissue damage was scored using a modified semi‐quantitative histopathological scoring system adapted from Li et al. (2014). The scoring system included epithelial loss, cytoplasmic vacuolation, and glandular dilatation, which were the principal histopathological findings observed in the gastric mucosa. Each parameter was assigned a score ranging from 0 to 3 (0 = normal, 1 = mild, 2 = moderate, and 3 = severe). The total histopathological score was obtained by summing the scores of the three parameters. All histopathological assessments were performed by a single investigator who was blinded to the treatment groups.

### 2.6 Immunohistochemical Examination

Apoptotic activity was assessed by immunohistochemical staining for Bax and Bcl‐2. Paraffin‐embedded gastric tissue sections (5 μm) were deparaffinized in xylene and rehydrated through graded ethanol solutions. Antigen retrieval was carried out in citrate buffer (pH 6.0) using microwave heating for 15–20 min. Endogenous peroxidase activity was blocked with 3% H_2_O_2_ for 10 min. The sections were then incubated with Ultra V Block to reduce nonspecific binding.

Primary antibody incubation was performed overnight at 4°C using anti‐Bax antibody (Affinity Biosciences, Cat. No. AF0120; 1:200) and anti‐Bcl‐2 antibody (Affinity Biosciences, Cat. No. AF6139; 1:200). After incubation with HRP‐conjugated secondary antibodies, immunoreactivity was detected using 3,3′‐diaminobenzidine (DAB). The sections were counterstained with hematoxylin.

Immunohistochemical staining was evaluated according to the method described by Qiao et al. (2011). Cells exhibiting brown cytoplasmic immunoreactivity were considered positive for Bax or Bcl‐2. For each rat, positive cells were counted in 10 randomly selected microscopic fields, and the percentage of positive cells was calculated as follows:

$$\text{Positive cells}\;\left(\%\right)=\left(\text{positive cells}/\text{total cells}\right)\times 100$$

All slides were examined by a single investigator who was blinded to the experimental groups using a light microscope (Carl Zeiss Axiolab 5, Carl Zeiss, Oberkochen, Germany). Image analysis and scale calibration were performed using ImageJ software (version 1.53; National Institutes of Health, Bethesda, MD, USA).

### 2.7 Evaluation of DNA Damage by the Comet Assay

DNA damage in gastric tissue was evaluated using the neutral comet assay according to the method reported by Bakır et al. (2026), with minor modifications. Approximately 0.5 g of gastric tissue was finely minced with a sterile scalpel and transferred into 2 mL of ice‐cold PBS. The tissue suspension was mixed at 500 rpm for 5 min and then kept on ice for 10 min. The cell‐containing supernatant obtained from minced gastric tissue was used for all comet assay procedures. Cell viability was not assessed before slide preparation, and no viability‐based exclusion procedure was applied.

Microscope slides were first coated with 0.5% normal‐melting‐point agarose and left to dry overnight at room temperature. A volume of 100 μL of the cell‐containing supernatant was then mixed with 0.8% low‐melting‐point agarose and immediately spread onto the precoated slides. The slides were covered with coverslips and placed on ice for 10 min to allow the agarose to solidify. After removal of the coverslips, the slides were immersed for 4 min at 4°C in freshly prepared cold lysis buffer containing 25 g of sodium dodecyl sulfate (SDS) dissolved in 1 L of Tris–borate–EDTA (TBE) buffer, following the protocol described by Bakır et al. (2026) and Yavaş et al. (2026).

Following lysis, the slides were transferred to a horizontal electrophoresis chamber containing freshly prepared electrophoresis buffer (TBE: 54‐g Tris, 27.5‐g boric acid, and 20‐mL EDTA; pH 8.4). DNA was allowed to unwind for 10 min before electrophoresis. Electrophoresis was carried out at 64 V and 250 mA for 2 min at 4°C. The slides were subsequently rinsed with distilled water for 5 min, neutralized, and stained with 50 μL ethidium bromide (1 μg/mL) before coverslips were applied.

To protect the fluorescent dye from light exposure, all staining and imaging procedures were performed under dark conditions. The slides were examined under a fluorescence microscope (Olympus BX51, Tokyo, Japan) at 400× magnification. For each animal, 50 randomly selected cells were analyzed using the Comet Assay Software Project (CASP, version 1.2.2). The evaluated comet parameters included head length, tail length, comet length, head DNA (%), tail DNA (%), tail moment (TM), and olive tail moment (OTM). Cells showing a typical comet pattern caused by DNA migration were considered to exhibit DNA damage.

### 2.8 Statistical Analysis

Statistical analyses were conducted using IBM SPSS Statistics for Windows, Version 26.0 (IBM Corp., Armonk, NY, USA). Data normality was evaluated using the Shapiro–Wilk test, and homogeneity of variance was assessed using Levene's test. Continuous variables are presented as mean ± standard deviation (SD) when normally distributed and as median (interquartile range [IQR]) when non‐normally distributed. Comparisons among groups were performed using one‐way ANOVA for normally distributed variables with homogeneous variances, followed by Tukey's honestly significant difference (HSD) post hoc test. When the homogeneity of variance assumption was not satisfied, Welch's ANOVA followed by the Games–Howell post hoc test was used. Histopathological scores were analyzed using the Kruskal–Wallis test. When the Kruskal–Wallis test indicated a significant difference, pairwise comparisons were performed using Dunn's test with Bonferroni correction. Dose–response relationships were examined using polynomial contrast analysis based on one‐way ANOVA. The analysis included tests for the overall group effect, linear trend, and deviation from linearity. To account for the evaluation of multiple outcomes, the overall group‐comparison *p* values were adjusted using the Benjamini–Hochberg false discovery rate procedure. All statistical tests were two‐sided. A *p* value < 0.05 was considered statistically significant for the primary analyses, and an adjusted *p* value (*q* value) < 0.05 was considered statistically significant in the false discovery rate analysis.

## 3 Results

### 3.1 Inflammatory, Oxidative Stress, and Gastric Mucosal Biomarkers

DEP administration significantly altered inflammatory, oxidative stress, and gastric mucosal biomarkers (Table 1).

**TABLE 1:** Effects of DEP exposure on inflammatory, oxidative stress, and gastric mucosal biomarkers.

Parameter	Control	DEP‐100	DEP‐300	DEP‐600	*p*
IL‐6 (ng/mL)	1.65 ± 0.17	1.75 ± 0.17	2.02 ± 0.09^a^, ^b^	2.50 ± 0.17^a^, ^b^, ^c^	< 0.001
TNF‐α (ng/mL)	1.79 ± 0.09	2.01 ± 0.10^a^	2.28 ± 0.05^a^, ^b^	2.52 ± 0.13^a^, ^b^, ^c^	< 0.001
SOD (ng/mL)	13.10 ± 0.33	11.75 ± 0.34^a^	9.18 ± 0.61^a^, ^b^	8.96 ± 0.21^a^, ^b^	< 0.001
MDA (nmol/mL)	5.04 ± 0.19	9.06 ± 0.25^a^	10.65 ± 0.60^a^, ^b^	12.53 ± 0.24^a^, ^b^, ^c^	< 0.001
COX‐2 (ng/mL)	4.38 ± 0.05	4.45 ± 0.01^a^	4.57 ± 0.06^a^, ^b^	4.64 ± 0.08^a^, ^b^	< 0.001
TFF1 (ng/mL)	3.14 ± 0.34	3.72 ± 0.19^a^	3.39 ± 0.21	2.72 ± 0.31^a^, ^b^, ^c^	< 0.001
MUC5AC (ng/mL)	2.21 ± 0.09	2.54 ± 0.07^a^	2.31 ± 0.30	1.93 ± 0.04^a^, ^b^	< 0.001

*Note:* Values are presented as mean ± SD (*n* = 7 animals per group). One‐way ANOVA followed by Tukey's HSD post hoc test was used for IL‐6, TNF‐α, SOD, and TFF1; Welch's ANOVA followed by the Games–Howell post hoc test was used for MDA, COX‐2, and MUC5AC. Superscript letters indicate significant pairwise differences.

Abbreviations: COX‐2, cyclooxygenase‐2; IL‐6, interleukin‐6; MDA, malondialdehyde; MUC5AC, mucin 5AC; SOD, superoxide dismutase; TFF1, trefoil factor 1; TNF‐α, tumor necrosis factor‐α.

^a^ *p* < 0.05 versus control.

^b^ *p* < 0.05 versus DEP 100 mg/kg.

^c^ *p* < 0.05 versus DEP 300 mg/kg.

IL‐6 levels were significantly higher in the 300 and 600 mg/kg groups than in the control and 100 mg/kg groups (all *p* < 0.05). TNF‐α levels increased significantly in all DEP‐treated groups compared with the control group, with further increases observed in the 300 and 600 mg/kg groups compared with the 100 mg/kg group (all *p* < 0.05). SOD levels were significantly reduced in all DEP‐treated groups relative to controls and were further decreased in the DEP‐300 and DEP‐600 groups compared with the DEP‐100 group (all *p* < 0.05). MDA levels differed significantly among all experimental groups according to the Games–Howell post hoc test (all *p* < 0.05). Similarly, COX‐2 levels were significantly elevated in all DEP‐treated groups compared with controls and were higher in the 300 and 600 mg/kg groups than in the 100 mg/kg group (Games–Howell, all *p* < 0.05).

TFF1 levels increased significantly in the 100 mg/kg group compared with controls but were significantly lower in the 600 mg/kg group than in the control, 100 mg/kg, and 300 mg/kg groups (all *p* < 0.05). Likewise, MUC5AC levels were significantly increased in the 100 mg/kg group compared with controls, whereas they were significantly lower in the 600 mg/kg group than in the control and 100 mg/kg groups. No significant difference was observed between the 300 and 600 mg/kg groups (Games–Howell, *p* = 0.055).

### 3.2 Histopathological Findings

Histopathological evaluation of hematoxylin and eosin (H&E)‐stained gastric tissue sections demonstrated dose‐dependent gastric mucosal injury following DEP exposure (Figure 1a–d). The control group exhibited normal gastric histoarchitecture, characterized by an intact surface epithelium and well‐organized gastric glands (Figure 1a). In the DEP‐100 group, mild histopathological alterations were observed, including focal superficial epithelial loss and mild cytoplasmic vacuolation of mucosal epithelial cells, while the deeper glandular architecture remained largely preserved (Figure 1b). In the DEP‐300 group, gastric mucosal injury became more pronounced, with evident epithelial desquamation, marked cytoplasmic vacuolation, focal cellular degeneration, and early glandular dilatation (Figure 1c). In the DEP‐600 group, severe and extensive gastric mucosal damage was observed, characterized by widespread epithelial loss, mucosal erosion, diffuse glandular dilatation, and marked disruption of the normal gastric architecture (Figure 1d).

**FIGURE 1:**
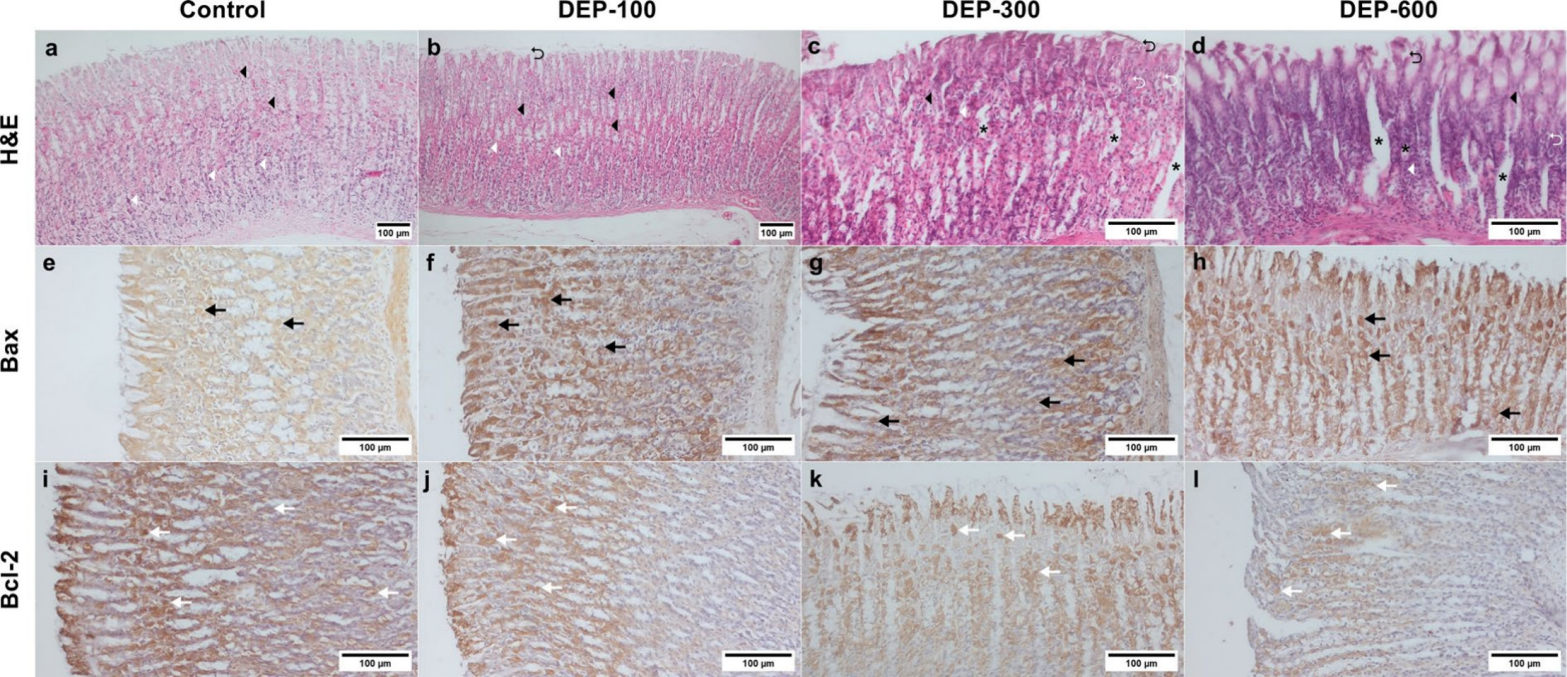
Microphotographs of gastric tissues from the experimental groups. Top row (a–d): H&E staining showing dose‐dependent histopathological changes. The control group showed normal gastric histology. In the DEP groups, superficial epithelial loss (black curved arrows), cytoplasmic vacuolation (white curved arrows), cellular degeneration (hash symbols), and glandular dilatation (asterisks) were observed. Parietal cells (black arrowheads) and chief cells (white arrowheads) are also indicated. Middle row (e–h): Bax immunohistochemical staining; brown cytoplasmic staining is seen (black arrows). Bottom row (i–l): Bcl‐2 immunohistochemical staining; brown cytoplasmic staining is seen (white arrows). Panels a and b were captured at ×100 magnification, whereas panels c–l were captured at ×200 magnification. Scale bars = 100 μm.

Semi‐quantitative histopathological scoring confirmed significant differences among the experimental groups (Figure 2). Epithelial loss differed significantly among the groups (*H* = 19.895, *p* < 0.001), with median scores increasing from 0 (0–0) in the control group to 1 (1–2), 2 (1–3), and 2 (2–3) in the DEP‐100, DEP‐300, and DEP‐600 groups, respectively (Figure 2A). Cytoplasmic vacuolation also increased significantly (*H* = 22.830, *p* < 0.001), with median scores of 0 (0–0), 1 (1–1), 2 (2–2), and 3 (2–3) across the respective groups (Figure 2B). Likewise, glandular dilatation increased progressively following DEP administration (*H* = 22.882, *p* < 0.001), with median scores of 0 (0–0), 0 (0–1), 1 (1–2), and 3 (2–3) in the control, DEP‐100, DEP‐300, and DEP‐600 groups, respectively (Figure 2C). Consistent with these findings, the total histopathological score increased significantly in a dose‐dependent manner (*H* = 24.243, *p* < 0.001), rising from 0 (0–0) in the control group to 3 (1–3), 6 (5–6), and 8 (6–9) in the DEP‐100, DEP‐300, and DEP‐600 groups, respectively (Figure 2D).

**FIGURE 2:**
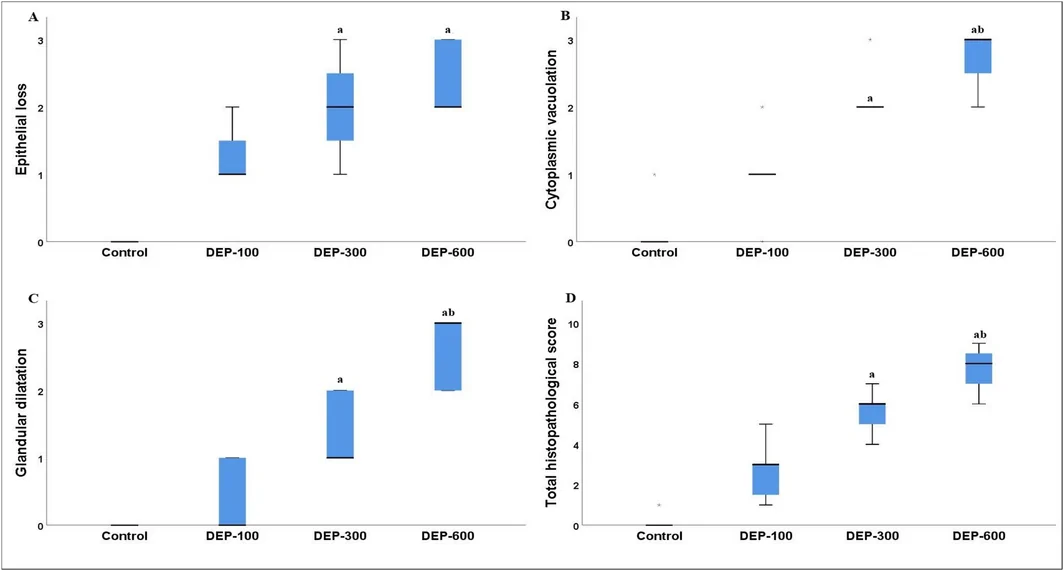
Histopathological injury scores in gastric tissue following DEP exposure. (A) Epithelial loss, (B) cytoplasmic vacuolation, (C) glandular dilatation, and (D) total histopathological score. Data are presented as boxplots (*n* = 7 animals per group). Pairwise comparisons were performed using Dunn's test with Bonferroni correction. ^a^ *p <* 0.05 versus Control; ^b^ *p <* 0.05 versus DEP‐100.

### 3.3 Immunohistochemical Findings

In the control group, Bax immunoreactivity was minimal, appearing as weak and sparse brown cytoplasmic staining in mucosal cells (Figure 1e). Following DEP administration, a dose‐dependent increase in Bax expression was observed. The number of Bax‐positive cells and staining intensity increased moderately in the DEP‐100 group (Figure 1f), became widespread throughout the gastric glands in the DEP‐300 group (Figure 1g), and showed intense, diffuse cytoplasmic staining in the DEP‐600 group (Figure 1h).

In contrast, Bcl‐2 immunoreactivity was strong and diffusely distributed throughout the gastric mucosa in the control group (Figure 1i). DEP exposure resulted in a dose‐dependent reduction in Bcl‐2 expression. Staining intensity was partially reduced in the DEP‐100 group (Figure 1j), became confined to focal areas in the DEP‐300 group (Figure 1k), and was reduced to minimal levels in the remaining gastric mucosa in the DEP‐600 group (Figure 1l).

The percentage of Bax‐positive cells increased significantly with increasing DEP dose, whereas the percentage of Bcl‐2‐positive cells decreased progressively (both *p* < 0.001). Consequently, the Bax/Bcl‐2 ratio increased markedly in a dose‐dependent manner and reached its highest value in the DEP‐600 group (*p* < 0.001; Table 2).

**TABLE 2:** Effects of DEP exposure on apoptotic biomarkers in gastric tissue.

Parameter	Control	DEP‐100	DEP‐300	DEP‐600	*p*
Bax (%)	8.19 ± 1.96	26.14 ± 2.74^a^	56.45 ± 4.14^a^, ^b^	87.02 ± 4.80^a^, ^b^, ^c^	< 0.001
Bcl‐2 (%)	82.04 ± 3.81	57.36 ± 5.32^a^	31.21 ± 3.82^a^, ^b^	10.98 ± 3.39^a^, ^b^, ^c^	< 0.001
Bax/Bcl‐2 ratio	0.10 ± 0.03	0.46 ± 0.05^a^	1.83 ± 0.26^a^, ^b^	8.62 ± 2.63^a^, ^b^, ^c^	< 0.001

*Note:* Values are presented as mean ± standard deviation (SD; *n* = 7 animals per group). Groups were compared using one‐way analysis of variance (ANOVA), followed by the Games–Howell post hoc multiple comparison test.

Abbreviations: Bax, Bcl‐2‐associated X protein; Bax/Bcl‐2 ratio, ratio of Bax to Bcl‐2 protein expression; Bcl‐2, B‐cell lymphoma 2.

^a^ *p* < 0.05 versus the control group.

^b^ *p* < 0.05 versus the 100 mg/kg DEP group.

^c^ *p* < 0.05 versus the 300 mg/kg DEP group.

### 3.4 Comet Assay Findings

DEP exposure induced significant alterations in all comet assay parameters (Table 3, Figure 3). L. Head values were significantly higher in the DEP‐300 and DEP‐600 groups than in the Control and DEP‐100 groups (all *p* < 0.05), whereas no significant difference was observed between the DEP‐300 and DEP‐600 groups (*p* > 0.05). Games–Howell post hoc analysis demonstrated significant differences among all experimental groups for L. Tail and L. Comet (all *p* < 0.05). Head DNA (%) decreased progressively with increasing DEP dose, whereas Tail DNA (%) increased significantly, with Tukey's post hoc analysis showing significant differences among all groups (*p* < 0.05). Similarly, TM and OTM increased markedly in a dose‐dependent manner, and Games–Howell post hoc analysis demonstrated significant differences among all groups (all *p* < 0.05).

**TABLE 3:** Effects of DEP exposure on comet assay parameters in gastric tissue.

Parameter	Control	DEP‐100	DEP‐300	DEP‐600	*p*
L.Head	232.32 ± 12.52	264.88 ± 12.94^a^	329.20 ± 20.38^a^, ^b^	313.74 ± 15.42^a^, ^b^	< 0.001
L.Tail	46.81 ± 9.75	126.25 ± 6.99^a^	246.17 ± 14.70^a^, ^b^	418.10 ± 20.42^a^, ^b^, ^c^	< 0.001
L.Comet	279.12 ± 20.92	391.31 ± 12.43^a^	571.90 ± 22.39^a^, ^b^	726.70 ± 40.27^a^, ^b^, ^c^	< 0.001
Head DNA(%)	95.80 ± 0.42	85.66 ± 1.17^a^	74.18 ± 1.05^a^, ^b^	65.66 ± 1.05^a^, ^b^, ^c^	< 0.001
Tail DNA (%)	4.20 ± 0.42	14.34 ± 1.17^a^	25.82 ± 1.05^a^, ^b^	34.30 ± 1.01^a^, ^b^, ^c^	< 0.001
Tail moment	1.98 ± 0.41	16.19 ± 1.64^a^	56.08 ± 6.57^a^, ^b^	130.70 ± 15.58^a^, ^b^, ^c^	< 0.001
Olive tail moment	3.81 ± 0.57	16.63 ± 1.63^a^	43.64 ± 5.88^a^, ^b^	85.67 ± 10.33^a^, ^b^, ^c^	< 0.001

*Note:* Values are presented as mean ± SD (*n* = 7 animals per group). L. Head, Head DNA, and Tail DNA were analyzed using one‐way ANOVA followed by Tukey's HSD post hoc test. L. Tail, L.Comet, tail moment (TM), and olive tail moment (OTM) were analyzed using Welch's ANOVA followed by Games–Howell multiple comparisons because the assumption of homogeneity of variances was not satisfied. Superscript letters indicate significant pairwise differences.

Abbreviations: Head DNA, percentage of DNA in the comet head; L. Comet, comet length; L. Head, head length; L. Tail, tail length; OTM, olive tail moment; Tail DNA, percentage of DNA in the comet tail; TM, tail moment.

^a^ *p* < 0.05 versus control.

^b^ *p* < 0.05 versus DEP 100 mg/kg.

^c^ *p* < 0.05 versus DEP 300 mg/kg.

**FIGURE 3:**
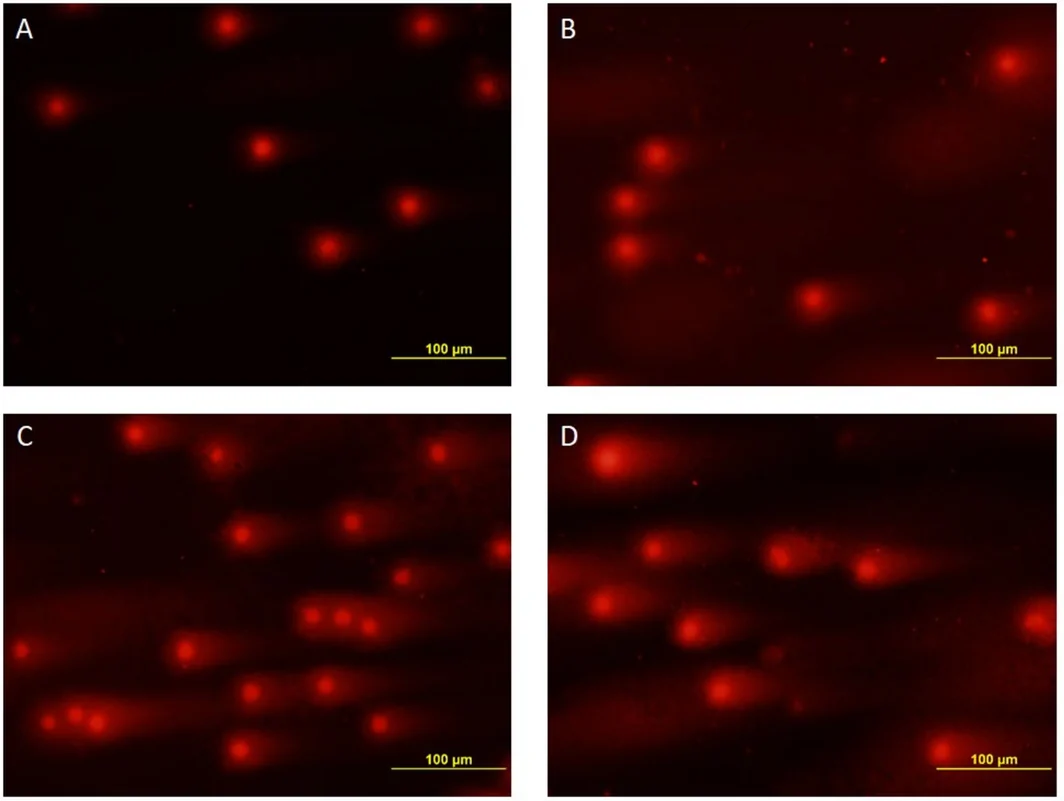
Representative neutral comet assay images of cells obtained from gastric tissue. (A) Control; (B) DEP 100 mg/kg; (C) DEP 300 mg/kg; and (D) DEP 600 mg/kg. The mean Tail DNA values for the corresponding groups were 4.20%, 14.34%, 25.82%, and 34.30%, respectively. Ethidium bromide staining, ×400 (Olympus, Japan).

### 3.5 Polynomial Trend Analysis

The results of the polynomial contrast analysis performed to evaluate dose–response relationships are presented in Table 4. Among the inflammatory, oxidative stress, and gastric mucosal biomarkers, IL‐6, SOD, MDA, TFF1, and MUC5AC exhibited significant linear trends together with significant deviations from linearity, indicating non‐linear dose–response relationships. In contrast, TNF‐α and COX‐2 showed significant linear trends without significant deviations from linearity.

**TABLE 4:** Polynomial trend analysis of biochemical, comet assay, and immunohistochemical parameters following DEP exposure.

Parameter	Overall ANOVA (F)	Overall *p*	Linear trend (*F*)	Linear *p*	Deviation from linearity (*F*)	Deviation *p*
IL‐6 (ng/mL)	43.895	< 0.001	121.398	< 0.001	5.144	0.014
TNF‐α (ng/mL)	77.116	< 0.001	230.880	< 0.001	0.234	0.793
SOD (ng/mL)	176.682	< 0.001	488.076	< 0.001	20.986	< 0.001
MDA (nmol/mL)	555.400	< 0.001	1583.009	< 0.001	41.596	< 0.001
COX‐2 (ng/mL)	27.759	< 0.001	82.020	< 0.001	0.629	0.542
TFF1 (ng/mL)	16.794	< 0.001	11.848	0.002	19.267	< 0.001
MUC5AC(ng/mL)	17.012	< 0.001	15.164	0.001	17.936	< 0.001
L.Head	56.915	< 0.001	136.410	< 0.001	17.168	< 0.001
L.Tail	940.203	< 0.001	2743.288	< 0.001	38.660	< 0.001
L.Comet	401.931	< 0.001	1196.536	< 0.001	4.628	0.020
Head DNA (%)	1302.971	< 0.001	3897.041	< 0.001	5.936	0.008
Tail DNA (%)	1327.909	< 0.001	3971.207	< 0.001	6.260	0.006
Tail moment	322.907	< 0.001	879.887	< 0.001	44.417	< 0.001
Olive tail moment	254.242	< 0.001	721.298	< 0.001	20.714	< 0.001
Bax‐positive cells (%)	653.304	< 0.001	1934.316	< 0.001	12.799	< 0.001
Bcl‐2‐positive cells (%)	389.124	< 0.001	1164.251	< 0.001	1.561	0.231
Bax/Bcl‐2 ratio	63.340	< 0.001	144.879	< 0.001	22.570	< 0.001

*Note:* Polynomial contrast analysis was performed using one‐way ANOVA to characterize dose–response trends across the ordered DEP exposure levels. The linear contrast tests for an ordered linear component across increasing DEP doses, whereas the deviation from linearity assesses whether the observed response departs from a purely linear pattern. A significant deviation from linearity indicates a non‐linear dose–response relationship. All overall group differences remained significant after Benjamini–Hochberg false discovery rate correction (all *q* < 0.001).

Abbreviations: ANOVA, analysis of variance; Bax, Bcl‐2‐associated X protein; Bcl‐2, B‐cell lymphoma 2; COX‐2, cyclooxygenase‐2; DEP, diethyl phthalate; DNA, deoxyribonucleic acid; IL‐6, interleukin‐6; L. Comet, comet length; L. Head, head length; L. Tail, tail length; MDA, malondialdehyde; MUC5AC, mucin 5AC; SOD, superoxide dismutase; TFF1, trefoil factor 1; TNF‐α, tumor necrosis factor‐α.

All comet assay parameters demonstrated significant linear trends. In addition, significant deviations from linearity were observed for L. Head, L. Tail, L. Comet, Head DNA (%), Tail DNA (%), TM, and OTM, indicating that the dose–response relationships were not entirely linear.

Similarly, Bax‐positive cells and the Bax/Bcl‐2 ratio exhibited significant linear trends together with significant deviations from linearity, whereas Bcl‐2‐positive cells showed a significant linear trend without a significant deviation from linearity (Table 4).

## 4 Discussion

The present study evaluated the effects of different DEP doses on inflammation, oxidative stress, gastric mucosal protective factors, DNA damage, apoptosis, and histopathological alterations in the gastric mucosa using a single experimental model. The findings demonstrate that DEP induces dose‐related gastric toxicity, accompanied by progressive inflammatory, oxidative, genotoxic, and histopathological changes.

DEP exposure was associated with increased IL‐6, TNF‐α, and COX‐2 levels, indicating the development of a pronounced inflammatory response in the gastric mucosa. Gastric mucosal defense depends on the coordinated interaction of the mucus layer, gastric epithelium, and mucosal protective mechanisms (Breugelmans et al. 2022; Hoffmann 2020). Disruption of this protective barrier by environmental toxicants promotes the release of inflammatory mediators and contributes to the development of mucosal injury (Raish et al. 2021; Xu et al. 2023). Oxidative stress‐mediated activation of the NF‐κB signaling pathway enhances the production of pro‐inflammatory cytokines, including IL‐6 and TNF‐α, thereby sustaining the inflammatory response (Xu et al. 2023). Similarly, Wong et al. (2019) demonstrated that exposure to di(2‐ethylhexyl) phthalate (DEHP) increased COX‐2 expression in gastric adenocarcinoma cells. Consistent with these observations, our findings suggest that DEP exposure promotes inflammatory responses in the gastric mucosa, thereby contributing to gastric tissue injury.

Reduced SOD levels and elevated MDA levels demonstrate disrupted gastric cellular redox homeostasis following DEP exposure. Previous studies have shown that exposure to DEP and other phthalates can induce oxidative stress and inflammatory responses in different tissues (Weaver et al. 2020). Oxidative stress occurs when the generation of ROS exceeds the capacity of cellular antioxidant defense systems. Excessive ROS production promotes lipid peroxidation while impairing antioxidant defenses, ultimately disrupting redox homeostasis and aggravating oxidative injury (Ketsa et al. 2024). Experimental studies with other phthalate compounds have similarly demonstrated alterations in oxidative stress metabolism accompanied by changes in antioxidant defense systems and structural tissue damage (Aydemir et al. 2023). Consistent with these findings, our results suggest that inflammation and oxidative stress jointly contribute to DEP‐induced gastric mucosal injury.

DEP exposure induced dose‐related alterations in TFF1 and MUC5AC levels. Both factors increased at the lowest dose but decreased at the highest dose compared with controls, indicating a nonlinear response. The gastric mucus barrier is an important component of the defense system that protects the gastric epithelium from gastric acid, digestive enzymes, and luminal toxic agents (Breugelmans et al. 2022). TFF1 and MUC5AC support gastric mucosal defense through complementary functions. TFF1 contributes to epithelial repair and mucosal restitution, whereas MUC5AC, the major gel‐forming gastric mucin, forms the physical mucus barrier covering the epithelial surface (Breugelmans et al. 2022; Heuer et al. 2020; Hoffmann 2020). The increase observed at the lowest DEP dose may represent a compensatory response to early tissue injury. In contrast, the reduction in TFF1 and MUC5AC levels at the highest dose, together with the inflammatory and oxidative changes, may indicate weakening of gastric mucosal defense.

Progressive gastric mucosal injury was further supported by the increasingly pronounced histopathological alterations observed with increasing DEP doses, including epithelial loss, cytoplasmic vacuolation, and glandular dilatation. Disruption of gastric mucosal architecture is generally considered a consequence of inflammation‐ and oxidative stress‐mediated cellular injury (Raish et al. 2021). Similarly, experimental studies investigating other phthalate compounds have reported that increasing exposure levels are accompanied by progressively more pronounced degenerative histopathological changes associated with inflammation and oxidative stress (Aydemir et al. 2023). In the present study, the progressive worsening of histopathological alterations with increasing DEP doses, together with the reduction in TFF1 and MUC5AC levels at the highest dose, indicates that changes in mucosal protective factors were accompanied by structural tissue damage.

DEP exposure induced dose‐related alterations in comet assay parameters, indicating progressive DNA damage in gastric tissue with increasing DEP doses. Oxidative stress is considered one of the principal mechanisms underlying phthalate‐induced genotoxicity, whereby excessive production of ROS promotes oxidative DNA base modifications, single‐ and double‐strand DNA breaks, and genomic instability (Sicińska et al. 2021). Consistent with this mechanism, experimental studies of other phthalate compounds have demonstrated that increased ROS generation is closely associated with oxidative DNA damage and DNA strand breaks (Sicińska et al. 2021). In the present study, the progressive alterations observed in the comet assay parameters, together with the inflammatory, oxidative, and histopathological findings, suggest that DEP exposure is associated with genotoxic damage in gastric tissue. Because only the neutral comet assay was used, these findings indicate DNA strand damage but do not distinguish between single‐strand and double‐strand breaks.

When DNA damage exceeds cellular repair capacity, apoptotic processes may be initiated (Fu et al. 2016; Tripathi et al. 2019). In the present study, increasing DEP doses were associated with progressively increased Bax protein expression, decreased Bcl‐2 protein expression, and an elevated Bax/Bcl‐2 ratio, indicating a dose‐dependent shift in the apoptotic balance toward a pro‐apoptotic state in the gastric mucosa. Oxidative DNA damage and excessive reactive oxygen species production can disrupt the Bax/Bcl‐2 balance and promote apoptotic signaling (Fu et al. 2016; Tripathi et al. 2019). Similarly, experimental studies investigating other phthalate compounds have reported increased Bax expression, reduced Bcl‐2 expression, and enhanced apoptotic responses in association with oxidative stress (Chi et al. 2022; Fu et al. 2016; Tripathi et al. 2019). The concurrent increase in Bax protein expression, decrease in Bcl‐2 protein expression, and elevation of the Bax/Bcl‐2 ratio indicate a dose‐related shift toward apoptosis in the gastric mucosa. Experimental gastric injury models induced by ethanol or indomethacin have shown that mucosal damage is accompanied by increased lipid peroxidation and inflammatory cytokine production, reduced antioxidant activity and mucus‐dependent defense, and changes in apoptosis‐related proteins. In these models, more severe gastric injury was associated with increased MDA, TNF‐α, IL‐6, P53, or Bax and with reduced SOD, catalase, glutathione peroxidase, Bcl‐2, and mucus or glycoprotein production (Ahmed, Alqaisi, Ibrahim, et al. 2025; Ahmed, Alqaisi, Jabbar, et al. 2025; Al‐Qaisi et al. 2025). Conversely, interventions that preserved antioxidant activity and gastric mucus, reduced lipid peroxidation and inflammatory cytokines, and limited changes in Bax and Bcl‐2 were accompanied by lower lesion scores and less severe histopathological injury (Ahmed, Alqaisi, et al. 2026; Ahmed, Tawfeeq, et al. 2026; Alqaisi et al. 2026). Although these studies used chemically induced ulcer models, they reported oxidative, inflammatory, mucosal protective, apoptosis‐related, and histopathological changes similar to those observed following DEP exposure in the present study.

Although gene expression was not examined, the increased tissue levels of IL‐6, TNF‐α, and COX‐2 support the presence of gastric inflammation at the protein level. Changes in TFF1 and MUC5AC levels show that proteins involved in mucosal defense were also affected, while increased Bax, decreased Bcl‐2, and an elevated Bax/Bcl‐2 ratio indicate apoptosis‐related changes. These protein changes were accompanied by oxidative imbalance, DNA damage detected by the comet assay, and progressive histopathological injury. The findings show that these responses developed with increasing DEP exposure; however, their transcriptional basis and causal sequence could not be determined.

Sex may affect the toxic response to DEP, including responses in the gastrointestinal tract. In a dietary study involving male and female rats, DEP increased the absolute and relative weights of the stomach and small intestine in both sexes. These increases were statistically significant at all dietary concentrations in females, although histological examination did not show treatment‐related lesions (Brown et al. 1978). In another study comparing male and female Wistar rats, prolonged DEP exposure produced different changes in hepatic, renal, and serum biochemical parameters between the sexes, while liver histopathological alterations were observed in both (Sinkar and Rao 2007). The mechanisms responsible for these differences have not been examined in gastric tissue. Phthalate effects in female rodents may vary according to exposure period, dose, estrous stage, and sex‐steroid signaling (Adam and Mhaouty‐Kodja 2022). DEP itself has also shown weak estrogenic activity and modulation of estrogen receptor α‐related signaling in experimental systems (Fiocchetti et al. 2021; Harris et al. 1997). Whether these estrogen‐related mechanisms are involved in the gastric effects of DEP remains unknown.

This study has several limitations. First, the exposure conditions used in this study differ from typical human exposure to DEP. Humans are generally exposed to low DEP levels repeatedly through dermal contact with fragrances, cosmetics, and other consumer products, as well as through oral and inhalational routes. The use of personal care products, particularly perfumes and fragranced products, has been associated with higher urinary concentrations of monoethyl phthalate, the main metabolite of DEP (Parlett et al. 2013). In the present study, rats received DEP once daily by oral gavage for 21 days. Oral gavage delivers the entire daily dose directly to the gastrointestinal tract as a single bolus, whereas human exposure generally occurs intermittently through several routes. The absorption, systemic availability, and direct gastric exposure associated with gavage may therefore differ from those occurring in humans.

Using the body‐surface‐area conversion described in the US Food and Drug Administration guidance, with K_m_ values of 6 for rats and 37 for adult humans, the rat doses of 100, 300, and 600 mg/kg/day correspond to approximate human equivalent doses of 16.2, 48.6, and 97.3 mg/kg/day, respectively (U.S. Food and Drug Administration 2005). Even the lowest of these values is substantially higher than the conservative human exposure estimate of 0.73 mg/kg/day reported for dermal exposure to DEP from fragrances and cosmetic products (Api 2001). However, body‐surface‐area conversion does not account for differences in exposure route, duration, absorption, metabolism, toxicokinetics, repeated low‐level exposure, or tissue susceptibility. These human equivalent doses are therefore presented only for dose comparison and should not be considered expected human exposure levels, safety limits, or human toxicity thresholds.

The study was not designed to determine a no‐observed‐adverse‐effect level (NOAEL) or lowest‐observed‐adverse‐effect level (LOAEL) for gastric toxicity. Because 100 mg/kg/day was the lowest dose tested and changes in several biochemical measurements were already present at this dose, a NOAEL could not be identified. However, these biochemical changes were not sufficient to define 100 mg/kg/day as a formal LOAEL. No adverse‐effect threshold was specified before the study, and some of the changes may represent adaptive responses of the gastric mucosa. In addition, the small number and wide spacing of the tested doses did not allow reliable threshold or benchmark‐dose estimation. The findings therefore describe gastric effects under short‐term, high‐dose gavage conditions and cannot be used to predict the effects of chronic low‐level human exposure. Further studies should include lower and more closely spaced doses, longer exposure periods, routes relevant to consumer exposure, and predefined criteria for adverse gastric effects.

Second, inflammatory, mucosal protective, and apoptosis‐related markers were evaluated at the protein level, but the corresponding gene expression and downstream signaling pathways were not examined. The results therefore do not demonstrate transcriptional regulation or establish the order or causal relationships among oxidative imbalance, inflammation, altered mucosal defense, DNA damage, and apoptosis. Studies combining RT‐qPCR analysis of *Il6*, *Tnf*, *Ptgs2*, *Tff1*, *Muc5ac*, *Bax*, and *Bcl2* with protein and pathway‐specific measurements are needed to examine these relationships.

Third, the fixed group size provided sufficient power to detect large effects but may have limited the detection of smaller differences among the groups. Fourth, cell viability was not assessed before the comet assay. DNA migration caused by mechanical tissue dissociation or nonviable cells therefore cannot be completely excluded. In addition, only the neutral comet assay was performed. Although this method is more sensitive to double‐strand DNA damage than the alkaline comet assay, it cannot reliably distinguish or separately quantify single‐strand and double‐strand breaks (Calini et al. 2002; Lu et al. 2017). The specific type of DNA strand damage observed in this study therefore remains uncertain. Further studies using complementary methods are needed to distinguish between these forms of DNA damage. Finally, only female Wistar rats were included, so possible sex‐related differences in gastric responses to DEP could not be evaluated, and the findings may not apply to males. Future studies should include male and female groups exposed to the same DEP doses for the same duration. The simultaneous evaluation of sex hormones, estrogen receptor‐related signaling, inflammation, oxidative changes, mucosal defense, DNA damage, apoptosis‐related markers, and histopathological injury would help determine whether gastric responses to DEP differ between sexes.

Our findings show that increasing DEP doses were accompanied by inflammatory and oxidative changes, altered gastric mucosal defense, structural tissue damage, DNA damage, and apoptosis‐related protein changes. These associated changes are summarized in Figure 4; however, their temporal and causal relationships require confirmation by gene expression and pathway‐specific analyses. The findings may provide a basis for further studies of DEP‐induced gastric toxicity and potential protective approaches.

**FIGURE 4:**
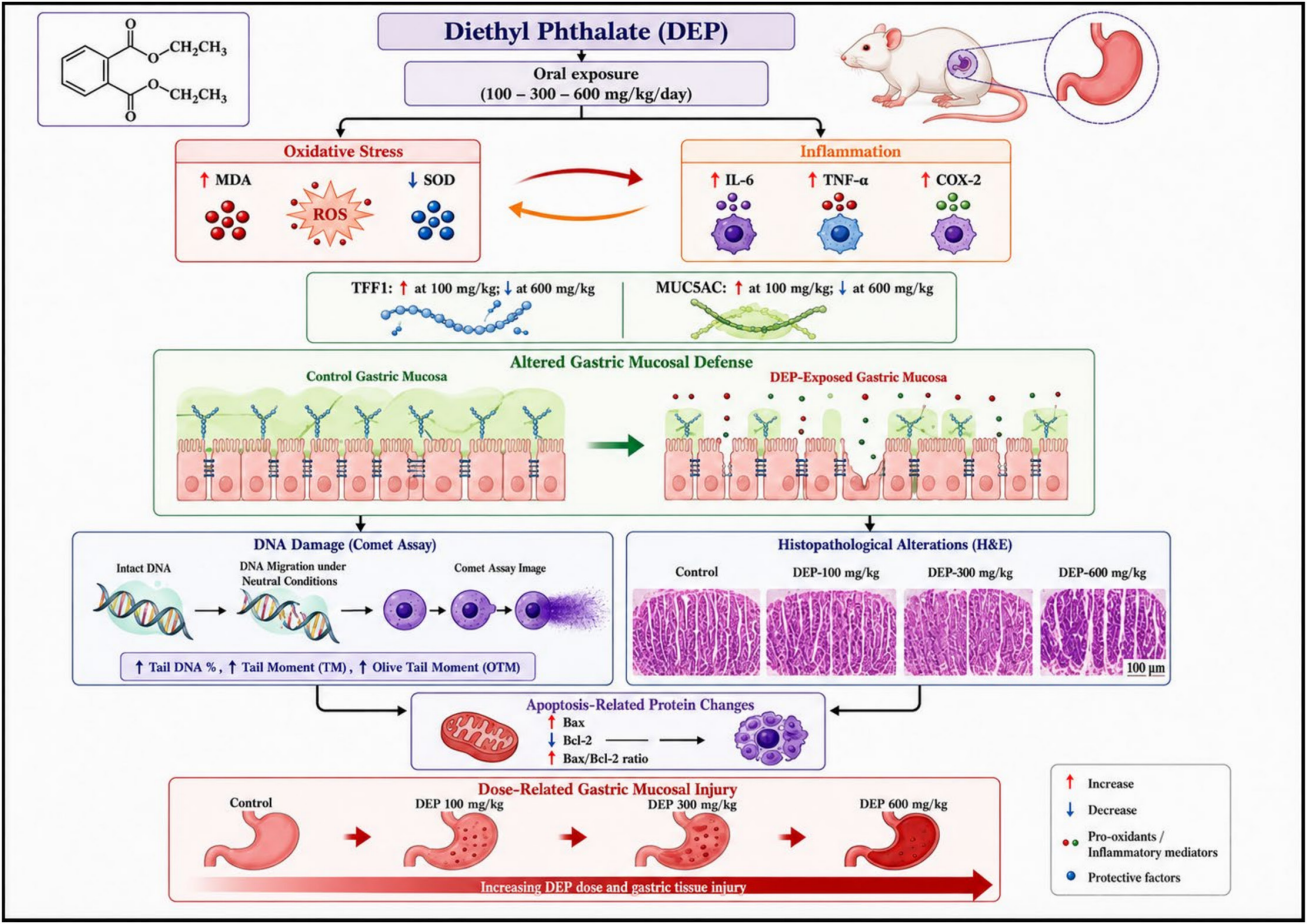
Schematic overview of the changes associated with DEP‐induced gastric toxicity. DEP exposure was associated with oxidative imbalance, inflammation, altered TFF1 and MUC5AC levels, changes in gastric mucosal defense, DNA damage, histopathological injury, and apoptosis‐related protein changes. The figure was conceptually organized with assistance from ChatGPT (OpenAI) and manually prepared and edited by the authors using Microsoft Paint 3D. All scientific content was reviewed and approved by the authors.

## Author Contributions

**Gul Sahika Gokdemir:** conceptualization, animal experiments, methodology, investigation, formal analysis, data curation, visualization, writing – original draft, writing – review and editing. **Mehmet Cihan Yavaş:** animal experiments, investigation, methodology, writing – review and editing. **Selim Demirtaş:** histopathological analysis, immunohistochemical analysis, investigation, writing – review and editing. **Fazile Cantürk Tan:** comet assay, investigation, writing – review and editing. **Salih Varol:** comet assay, investigation, writing – review and editing. **Mehmet Tahir Gokdemir:** conceptualization, supervision, validation, project administration, writing – review and editing.

## Funding

The authors have nothing to report.

## Ethics Statement

The original animal experiment was approved by the Local Ethics Committee for Animal Experiments of Dicle University Health Sciences Research and Application Center (Protocol No. 2024/34). Ethical approval for the use of previously unused gastric tissues from these animals in the present study was subsequently obtained from the same committee (Meeting No. 02, Decision No. 18; January 29, 2026). All procedures were conducted in accordance with applicable national regulations for laboratory animal care and the ARRIVE 2.0 guidelines.

## Conflicts of Interest

The authors declare no conflicts of interest.

## Data Availability

The datasets generated and/or analyzed during the current study are available from the corresponding author upon reasonable request.
